# Subtle Presentation of Two Rare Gynecological Cancers in a Single Patient: A Case Report

**DOI:** 10.7759/cureus.67239

**Published:** 2024-08-19

**Authors:** Eden Estevez, Frederick Friedman, Tiangui Huang, Nora Morgenstern

**Affiliations:** 1 Obstetrics and Gynecology, St. George’s University School of Medicine, St. George, GRD; 2 Obstetrics and Gynecology, Icahn School of Medicine at Mount Sinai, Elmhurst Hospital Center, Queens, USA; 3 Pathology, Icahn School of Medicine at Mount Sinai, Elmhurst Hospital Center, Queens, USA

**Keywords:** yolk sac tumor, human papillomavirus-independent cervical cancer, atypical glandular proliferation, gastric-type endocervical adenocarcinoma, cervical cancer, endocervical adenocarcinoma, gastric-type adenocarcinoma

## Abstract

Yolk sac tumors (YSTs) are rare germ-cell malignancies that usually develop in the gonads. Similarly, gastric-type adenocarcinoma of the endocervix (GAS) is a rare kind of gynecological cancer that has piqued interest due to its distinctive clinical and pathological features. These two malignancies in a single patient present a unique and challenging scenario. Here, we present the case of a 33-year-old female who presented with postcoital bleeding and was diagnosed with atypical glandular proliferation consistent with GAS. Interestingly, this patient had a history of a YST treated with left salpingo-oophorectomy and chemoradiation in the Philippines five years prior. A follow-up ultrasound report in the Philippines five months after treatment showed no evidence of residual disease. This case report aims to understand the predisposing factors of these neoplasms and asks if there is a link between them, which is necessary for tailoring surveillance, appropriate therapeutic approaches, and improving patient outcomes.

## Introduction

Yolk sac tumors (YSTs) are usually diagnosed in children and young adults under 40 [1]. These germ-cell tumors rapidly grow and tend to metastasize to lymph nodes early [1]. Treatment usually involves surgical removal and chemotherapy. Endocervical adenocarcinoma (ECA) is an uncommon type of cervical cancer that develops from the cervix's glandular cells and accounts for 20-25% of all cervical cancers [2]. We classify ECA into two main types based on histopathological features and etiological factors: human papillomavirus (HPV)-associated usual type and HPV-independent gastric type [3]. The literature has not extensively documented the uncommon occurrence of a YST and gastric-type adenocarcinoma of the cervix (GAS) in the same patient. This case report discusses the clinical presentation, diagnostic problems, and potential treatments for managing a patient with an unusual oncological history. We describe the case of a 33-year-old female with a prior history of YSTs, diagnosed and treated at the age of 28, who experienced postcoital bleeding and was diagnosed with HPV-negative glandular atypia. This report details a specific patient case to understand better the potential complexities and relationships between these rare and distinct malignancies.

## Case presentation

Herein, we have a 33-year-old G1P1001 female with a past medical history of a YST, status post left laparoscopic salpingo-oophorectomy and chemoradiation at age 28, who presented with postcoital bleeding and a sizeable vaginal mass with irregular contour on speculum examination. Magnetic resonance imaging (MRI) revealed a cervical mass. The patient has a family history of vaginal cancer, leukemia, and lung cancer.

**Figure 1:**
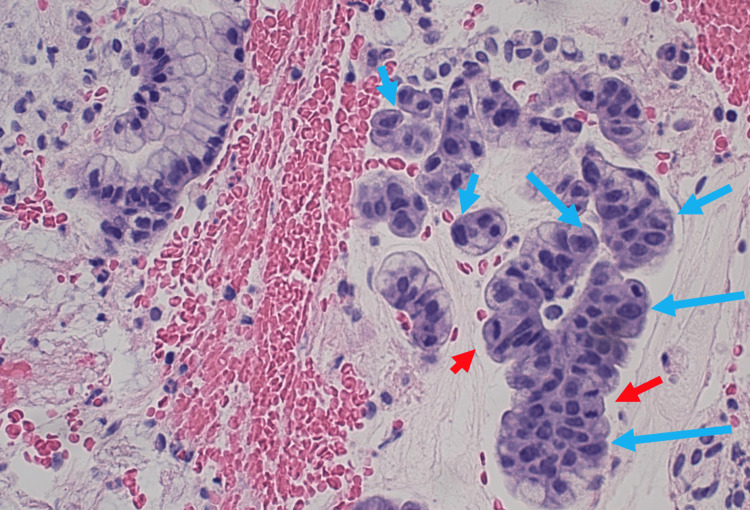
Atypical glandular proliferation later diagnosed as gastric-type ECA noted on the background of squamous metaplasia and unremarkable endocervical glands Red arrows: mitoses; blue arrows: nuclear atypia

**Figure 2:**
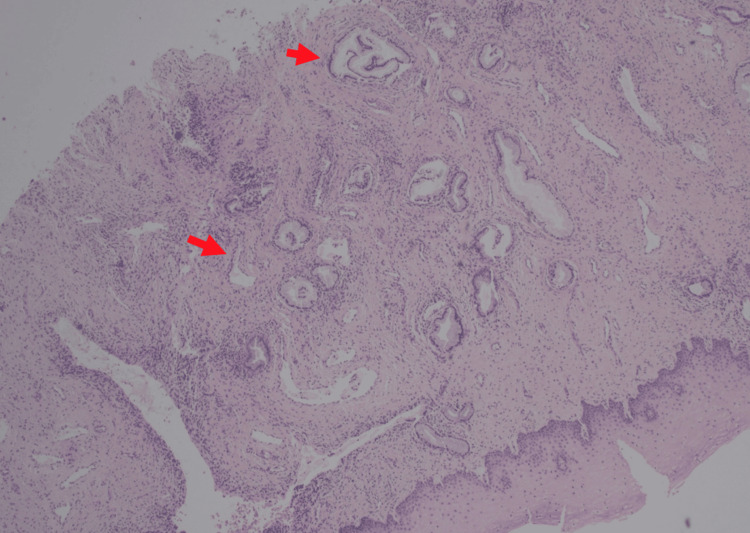
Cervicovaginal junction with irregularly sized and spaced glands infiltrating deep into the stroma

**Figure 3:**
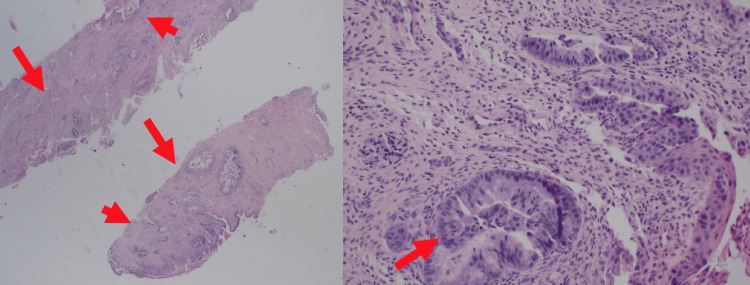
Anterior cervical biopsy with deeply infiltrating irregular glands (left) The detail of one gland shows an abrupt transition from bland cuboidal endocervical cells to tall hyperchromatic atypical endocervical cells (right)

An examination under anesthesia demonstrated a 2 cm ridge of tumor at the cervicovaginal junction circumferentially from 10 o’clock to 8 o’clock and a 0.5 cm ridge from 8 o’clock to 10 o’clock. The tumor mass was 5.5 cm wide by 6 cm anterior-posterior, with the predominant tumor on the anterior cervical lip. Endocervical and endometrial curettages revealed small glandular fragments with abundant mucin, some with increased mitoses and nuclear atypia (Figures 1-2). Nuclear atypia is seen as nuclei with varying abnormal shapes (triangular, rectangular, trapezoid) instead of the usual small round nuclei of benign cuboidal endocervical glands. There are varying nuclear sizes (some large and some small) instead of the more uniform size of benign endocervix. Some of the nuclei show nuclear membrane irregularities with notches and grooves. Some of the atypical nuclei appear hyperchromatic (Figure 3). There was also squamous atypia with increased Ki67 stains. The atypia was unusual since p16 stains were negative in both glandular and squamous components. Additional biopsies confirmed the presence of GAS.

**Figure 4:**
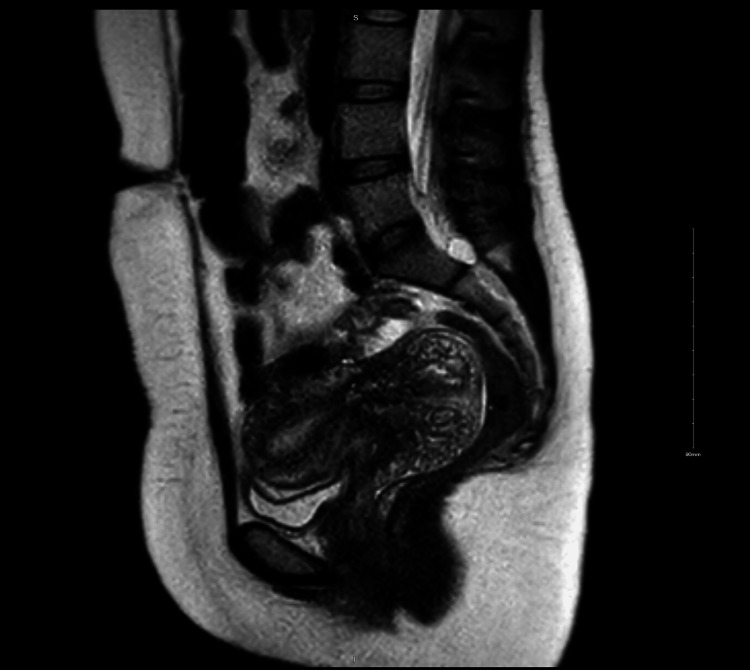
MRI T2-weighted image. Globular enlargement of the cervix containing multiple cystic spaces concerning adenoma malignum of the cervix, a variant of cervical mucinous adenocarcinoma

This patient’s tumor had abundant mucin, which manifested on MRI as a well-defined hyperintense mass on a T2-weighted image (Figure 4). MRI was able to provide an accurate determination of tumor size and location within the cervix.

The findings of this case were consistent with the International Federation of Gynecology and Obstetrics (FIGO) stage IIIB adenoma malignum of the cervix. Given her prior oncological history, gynecological oncology decided to refer the patient to a tertiary cancer treatment and research institution to devise an optimal management plan. She received chemoradiation and adjuvant chemotherapy treatment.

## Discussion

Social history and genetic factors influence a person's vulnerability to GAS and YSTs. Empirical evidence suggests a relationship between YST and the RB1 gene linked to retinoblastoma [4]. In addition, while the exact causation is unknown, the interplay of transcription factor GATA-4 and the RUNX3 gene has been postulated [5,6]. Moreover, YSTs are associated with elevated alpha-feto protein (AFP) levels, a marker for monitoring and diagnosis [7].

Approximately 10% of ECAs are not associated with an HPV infection [8]. Unlike HPV-associated adenocarcinomas, which have been declining in incidence, likely due to effective screening and HPV vaccination programs, the incidence of GAS appears to be stable or even increasing slightly [8]. The increase may be due to improved recognition and diagnostic techniques. Cells that resemble gastric mucosa characterize GAS, displaying pale or clear cytoplasm, distinct cell borders, and often a lobular pattern [8]. These tumors frequently have a diffuse growth pattern and may show signet-ring cell features [9].

GAS presents at more advanced stages due to a lack of symptoms in the early stages and the absence of HPV-related screening markers [2], which creates a barrier to diagnosis. It often shows deeper stromal invasion and a higher propensity for lymphovascular invasion and distant metastasis [8].

Due to its mucinous content, GAS usually presents as a well-defined hyperintense mass on T2-weighted images [10]. MRI provides an accurate determination of tumor size and location within the cervix [11]. MRI is highly effective in assessing the depth of stromal invasion and involvement of adjacent structures [11]. MRI can also detect enlarged pelvic and para-aortic lymph nodes and help identify metastasis to distant organs in particularly advanced cases [11].

The genetic disposition of GAS is less understood than that of YST. Certain factors such as chronic inflammation and genetic syndromes might affect the development. Predisposing genetic factors for GAS include Peutz-Jeghers Syndrome and Lynch Syndrome [12]. In contrast, YSTs are not typically associated with inherited cancer syndromes [1]. They arise sporadically, and there is no documentation in the literature to suggest that there are genetic predispositions like those in GAS [1].

While RB1 mutations and AFP levels significantly mark YST predisposition, genetic factors tied to GAS are still being studied. Immunohistochemical staining and molecular testing differentiate GAS from other cervical adenocarcinomas [13]. However, more research is needed to understand their pathogenesis and develop targeted treatments for these patients.

Treatment approaches for GAS are similar to those of other gynecological cancers such as YSTs, which include surgery, radiation therapy, and chemotherapy [9]. Still, the aggressive nature of GAS may call for more intensive treatment regimens and closer follow-up.

## Conclusions

This case report highlights the rare and complex encounter of a YST and GAS in one patient, even though we found no relation between the genetic predisposition of these two cancers. Gastric-type ECA is an uncommon and aggressive subtype of cervical cancer with unique clinical characteristics and a poor prognosis. Its incidence remains low but stable, and further research is required to understand its pathophysiology better and create more effective therapies. Given the patient's oncological history, a multidisciplinary approach to diagnosis and treatment is necessary. This case highlights the importance of considering rare gynecological malignancies in the differential diagnosis, developing customized treatment plans, and vigilant monitoring for these patients. Several syndromes predispose individuals to multiple malignancies; some not previously mentioned are Cowden Syndrome, Li-Fraumeni Syndrome, Fragile X Syndrome, and Von-Hippel-Lindau Syndrome. It is unclear if this case represents a new syndrome or undiagnosed tumor-related immunodeficiency different from the most notable ones.
